# Antibiotic utilization in outpatient and inpatient hospitals in Zambia: a systematic review, key findings and public health implications

**DOI:** 10.1016/j.infpip.2026.100547

**Published:** 2026-04-23

**Authors:** L. Siachalinga, N. Nyirenda, M. Hachalinga, R.K. Mutati, W. Mufwambi, A.C. Kalungia, B. Godman, H. Kim

**Affiliations:** a School of Medicine and Dentistry, Griffith University, 58 Parklands Drive, Southport, Queensland, Australia; b Department of Minority Health and Health Disparities Research Georgetown University, Washington, DC, USA; c Zambia Medicines Regulatory Authority, Department of Marketing Authorisation, Head Office, Plot No.2350/M, Off Kenneth Kaunda International Airport Road, Lusaka, Zambia; d Department of Pharmacy, School of Health Sciences, University of Zambia, P.O. Box 50110, Lusaka, Zambia; e Division of Public Health Pharmacy and Management, School of Pharmacy, Sefako Makgatho Health Sciences University, Pretoria, South Africa; f Antibiotic Policy Group, City St. George's, University of London, London, UK

**Keywords:** Antimicrobial utilization, Prevalence, Appropriateness, Quality indicators, Antimicrobial stewardship

## Abstract

This review aimed to document current antibiotic utilization patterns among Zambian hospitals to inform Antimicrobial Stewardship (AMS) policies and practices to help reduce AMR. Published literature was searched from PubMed, Embase and Google Scholar from 1 January 2000 to 13 March 2025. Search terms included antibiotics, utilization, intervention, stewardship and hospital while applying appropriate Boolean Operators. The review was conducted according to the preferred reporting items for systematic reviews and meta-analysis. The search retrieved 913 studies; 19 studies were included in the final analysis, including 17 quantitative descriptive studies and two non-randomized controlled studies. The overall quality of the included studies was good (≥75%). Antibiotic use prevalence was between 50.3% - 82.5% with a pooled estimate of 67.0%, 95% CI (60–73). The highest prevalence were Access antibiotics (34.2%–84.0%). Frequently prescribed antibiotics belonged to the Watch group in 8 out of 11 studies. Two (10.5%) studies assessed antibiotic prescribing appropriateness, overall inappropriateness was 67.0% in one study, while the other study reported appropriateness over 95% across assessed indicators. In 10 studies that reported at least one quality indicator for antibiotic use, guideline compliance was the most reported with compliance ranging from 27.0% to 58.1%. Two studies that evaluated the impact of Antimicrobial Stewardship Programs (ASPs) on antibiotic use reported improvement in use pre to post ASP. In conclusion, despite structural challenges in diagnostics and guideline adherence, sustained multifaceted AMS implementation and integration of the WHO AWaRe framework are improving prescribing patterns and providing a pathway to curb antimicrobial resistance in Zambia.

## Background

Antimicrobial resistance (AMR) continues to be a global health concern and is increasingly considered as the next pandemic unless multiple activities are undertaken [1,2]. An estimated 4.9 million global deaths were attributed to AMR bacterial infections in 2019 [3,4], with the highest mortality rates seen among low- and middle-income countries (LMICs) including African countries [4,5]. AMR is driven by the misuse and overuse of antimicrobials across all sectors of healthcare, especially among LMICs [6,7], which if left unchecked would continue to appreciably increase AMR and associated mortality [5].

In recent years, multiple global, regional, and national initiatives have been instigated to improve future antimicrobial use and reduce AMR. The World Health Organization (WHO) launched the 2015 Global Action Plan (GAP) on AMR [8], which has subsequently been translated into National Action Plans (NAPs) [[9], [10], [11]]. However, the implementation of NAPs has faced multiple challenges among African countries due to limited resources and trained personnel [[11], [12], [13]]. Alongside this, the WHO in 2018 introduced the AWaRe (Access, Watch and Reserve) antibiotics classification as part of the Essential Medicines List to help reduce the use of Watch and Reserve antibiotics with their greater resistance potential [14]. The initial WHO target was that at least 60% of antibiotic use across sectors should be Access antibiotics [15]. The Access target has now increased to 70% following the United Nations General Assembly (UN GA) [16].

The GAP and NAPs emphasize monitoring of antibiotic utilization patterns through Point Prevalence Surveys (PPS) as a prerequisite to identify prescribing patterns of concern and guide targeted improvements [17,18]. Part of the suggested activities within NAPs are antibiotic stewardship programs (ASPs) which are key interventions to optimize antibiotic utilization across sectors among LMICs [[18], [19], [20]]. While ASPs have been considered challenging to instigate among LMICs due to limited resource, knowledge and personnel [21,22], this is changing as seen by a growing number of ASPs introduced across Africa including among hospitals [18,19,23,24]. These programs are increasingly guided by the WHO AWaRe utilization targets in LMICs, as well as the recent AWaRe guidance, reflecting wider adoption of the WHO AWaRe system and guidance to monitor antibiotic use and its integration into quality improvement targets given concerns with national antibiotic guidelines across LMICs [18,[25], [26], [27], [28]].

Zambia, like other African countries with high rates of AMR across sectors [[29], [30], [31], [32], [33]], has ongoing concerns with current limited active surveillance of AMR patterns, healthcare infrastructures, and knowledge of antibiotics and AMR among key stakeholders, including the AWaRe system, and limited ASP activities [29,[34], [35], [36], [37]]. Ongoing national activities to reduce inappropriate antibiotic use following the launch of the NAP include increased focus on AMR with capacity building [29,38,39]. Despite progress, concerns remain including high rates of Watch antibiotic utilization and limited implementation of ASPs [35,36,40].

Evidence on antibiotic utilization across Zambian hospitals is also currently fragmented. Addressing these gaps is important to guide key stakeholder across Zambia since hospitals, especially tertiary level, are training grounds for future healthcare professionals (HCPs). Exposed to suboptimal antibiotic prescribing practices, alongside limited AMS knowledge and activities, may translate into poor prescribing and dispensing habits once HCPs qualify and start treating patients in ambulatory care, which can account for up to 95% of total antibiotic use in humans [41,42].

Consequently, the aim of this study was to document current antibiotic utilization patterns across hospitals in Zambia including, prescribing indicators and ASPs given concerns of high levels of inappropriate use. The findings will support on going ongoing national efforts to improve future antibiotic prescribing, thereby reducing AMR and potentially provide direction to other African countries and LMICs facing similar challenges.

## Methods

This study was performed in accordance with the Preferred Reporting Items for Systematic Reviews and Meta-analysis (PRISMA) Checklist (Supplementary Table A1). The PRISMA checklist is an evidence-based tool for evaluating the title, abstract, methods, results, discussion and findings. It can be used for evaluating randomized controlled trials and reporting systematic reviews for non-heterogeneous research [43]. There was no protocol registered for this review.

### Search strategy

The primary databases for our search were PubMed and Embase. Google Scholar was added to capture relevant literature not indexed in traditional databases, such as more recent publications. The review included all eligible studies from Zambia published from 1^st^ January 2000 to 13^th^ March 2025. The search terms were a combination of the following keywords, (antibiotic OR antimicrobial) AND (use OR utilization OR interventions OR antimicrobial stewardship OR program) AND (hospital) AND (Zambia), see Supplementary Table A2. Embase subject headings (Emtree) and medical subject headings (MeSH) were applied accordingly. No restrictions in study design and language were applied. A filter was applied to exclude studies focusing on tuberculosis and human immunodeficiency virus (HIV).

### Study selection

All studies documenting antibiotic utilization patterns and interventions to improve antibiotic use in hospitals in Zambia were included in the review. All references were downloaded into Covidence [44] for screening. All studies were screened first according to title and then abstract and reviewed by two independent reviewers (LS and NN) for inclusion. Disagreements were resolved through discussions among the reviewers until consensus was reached. The selection process for eligible studies was based on a specified inclusion criterion (Table I).

**Table I:** Inclusion and exclusion criteria

	Inclusion criteria	Exclusion criteria
Study design	Randomized and non-randomized studies Published studies	Non-published studies Qualitative studies Reviews, case studies, abstracts, theses and study protocols Studies for which the full article was not accessible
Population	Hospital setting	Community settings Community pharmacies
Exposure	Reported the use of antibiotics	Studies in which no antibiotics use was reported
Outcomes	Antibiotic use prevalence Appropriateness of antibiotics use Quality indicators Interventions to improve antibiotic use	No outcome of interest Antimicrobial resistance

### Main outcomes of interest

The main outcomes of interest for our systematic review were current antibiotic utilization patterns, indicators and ASPs across Zambia. The utilization patterns included the prevalence of antibiotic use in hospitals, appropriateness of use, quality indicators employed to measure antibiotic use and any interventions implemented. Where possible, antibiotic utilization patterns were categorized by the AWaRe system [14].

### Quality assessment

The quality assessment of studies was conducted by two researchers (LS and NN) using the Mixed Method Appraisal Tool (MMAT) [45]. Each study was assessed using the MMAT criteria based on its methodological design. For each criterion, a score of 1 was applied if yes and 0 if no or unclear. The score for each study was determined by calculating the number of yeses out of the total number of applicable criteria. Studies were categorized as good (≥75%), moderate (50–75%), or poor quality (<50%) based on the proportion of criteria met. While we calculated the average score for all studies, we endeavoured to provide a more detailed presentation of the ratings of each criterion to better inform the quality of the included studies as recommended [45].

### Data extraction and analysis

Data extraction was performed using a standardized excel form by two reviewers (LS and MH). Extracted data included study first author, location (province), publication year, study design, objectives, and main outcomes. Descriptive analysis of findings was used to present findings. A random-effects meta-analysis was conducted in R version 4.4.2 to pool antibiotic use prevalence estimates across the included studies using the metaprop function [46]. Subgroup analysis was conducted based on study design, geographical location, health care setting or level and study population. Heterogeneity was assessed using I^2^ statistic and categorized as low < 25%, moderate 25–49%, substantial 50–74%, or high 75–100%, heterogeneity [47].

## Results

### Summary of included studies

**Figure 1:**
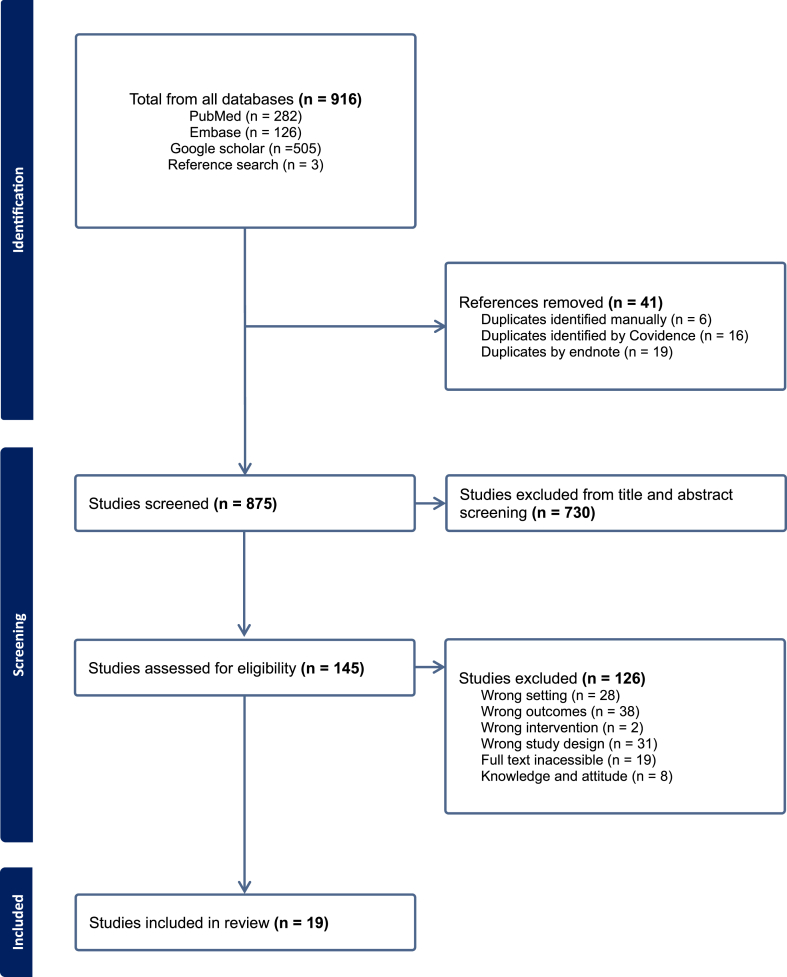
Prisma flow diagram.

**Figure 2:**
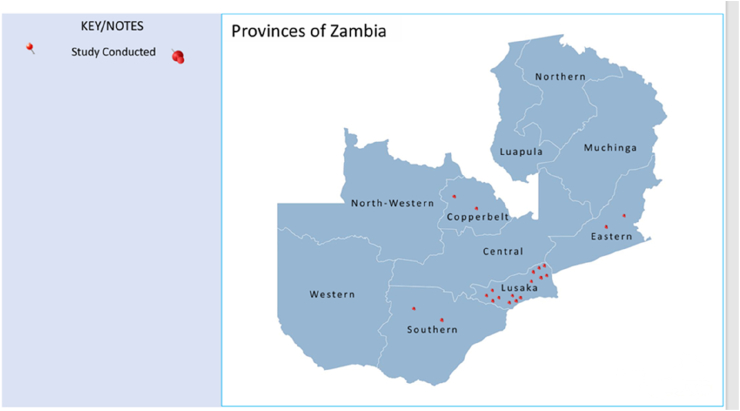
Geographic distribution of studies.

A total of 916 studies were identified, 913 from the three electronic databases and three from reference list screening. After removing duplicates as well as screening titles and abstracts, 145 studies were deemed eligible for full-text review. Of these, 19 subsequently met the inclusion criteria for the final analysis, Figure 1 presents the PRISMA flow diagram generated using Covidence [44]. A summary of the characteristics of the included studies is presented in Table II. Most of the included studies were observational cross-sectional in design, with only five being point prevalence studies [35,[48], [49], [50], [51], [52]]. Geographically, most studies were conducted in one province, with 11 from Lusaka Province alone [32,35,[52], [53], [54], [55], [56], [57], [58], [59], [60]], one in the Eastern Province at St. Francis Hospital [40], one in Southern Province [61]. Three studies included two provinces, one included Lusaka and Ndola [62], and the other two studies included Eastern province and Lusaka [49]. One study was conducted across Lusaka, Southern, and the Copperbelt provinces [63], and three studies were conducted in various hospitals across the country [48,50,64], see Figure 2 for the distribution of the studies across Zambia. In addition, while the studies by D'Arcy *et al.*, 2021 [49], Saleem *et al.*, 2025 [59] and Yasmin *et al.*, 2024 [60] were conducted across multiple countries, only data for Zambia was extracted and analyzed for this review. The studies were published between 2020 and 2025. Thirteen studies reported antibiotic use and AWaRe categorization [35,40,[48], [49], [50],^52^,^53^,[55], [56], [57], [58],^61^,^62^], two studies assessed the appropriateness of antibiotic prescribing [55,58], and 10 studies reported at least one quality indicator for antibiotic use [35,[48], [49], [50],^52^,[55], [56], [57], [58],^65^]. Only two studies evaluated the impact of an ASP on subsequent antibiotic use [64,65].

**Table II:** Summary of included studies

Author, year	Study design	Study period	Province	Health care level: - Population	Prevalence *n/N* (%)
Chizimu 2024 [48]	WHO-PPS	12/2023	Nationwide	Six tertiary, five secondary and five primary care hospitals: - inpatients (mixed∗)	908/1296 (70)
D'Arcy 2021 [49]	Global-PPS	05/2019–12/2019	Eastern, Lusaka	A tertiary and secondary care hospital: - inpatients (mixed)	238/418 (57)
Kalonga 2020 [53]	Cross-sectional	04/2019–07/2019	Lusaka	A tertiary care hospital: - in/outpatients (children)	281/357 (78.7)
Kalungia 2022 [50]	WHO-PPS	11/2021	Nationwide	10 primary and secondary care hospitals: - inpatients (mixed)	307/520 (59) Neonatal intensive care unit 83.3 mixed paediatric wards: 47.7 adult wards: 49.7 to 52.5
Kalungia 2024 [64]	Before and after	04/2022–04/2023	Nationwide	Two tertiary and eight secondary care hospitals: - inpatients (mixed)	Pre vs post int 740/1477 (50.1) vs 620/1400 (44.3)
Kasanga 2022 [54]	Cross-sectional	01/2020–12/2020	Lusaka	Two tertiary care hospitals: pre- caesarean section surgery use	836/838 (99.8)
Makiko 2025 [52]	Global PPS	07/2023–09/2023	Lusaka	Five primary care hospitals; - medical and surgical wards inpatients (mixed)	146/183 (79.8) Medical: 80 Surgical: 75
Masich 2020 [55]	Cross-sectional	06/2018	Lusaka	A tertiary care hospital: - adult inpatient of medical wards	88/146 (60)
Miyanda 2022 [56]	Cross-sectional	07/2018–05/2020	Lusaka	A primary care hospital: - adult inpatients of admission ward	290/385 (75.3)
Mudenda 2022 [35]	Cross-sectional	09/2021–11/2021	Lusaka	Five primary care hospitals adult in patients	320/388 (82.5)
Mudenda 2023 [58]	Cross-sectional	08/2021–09/2021	Lusaka	A tertiary care hospital: - in/outpatient (mixed)	NR
Mudenda 2024 [40]	Cross- sectional	08/2023–10/2023	Eastern	A secondary care hospital: - In/outpatients (mixed)	415/800 (51.9)
Mudenda 2025 [51]	WHO-PPS	08/2024	Lusaka	Five primary care hospitals: - inpatients (mixed)	342/580 (59) Female surgical ward: 79 (44/56) male medical ward: 76 (58/76) children's ward: 75 (70/93) male surgical ward: 74 (45/61) female medical ward: 61 (52/85) obstetrics and gynaecology: 35 (73/209)
Mudenda 2025 [61]	Cross-sectional	07/2022–06/2023	Southern	A primary care hospital: - outpatients (mixed)	600/302 (50.3)
Mudenda 2025 [63]	Before and after	08/2022, 10/2023	Lusaka, Southern, Copperbelt	Three tertiary care hospitals: - adult inpatients in medical and surgical wards	321/427 (75)
Saleem 2025 [59]	Prospective, observational	2020–2022	Lusaka	A tertiary care hospital: - pregnant women enrolled in the A-PLUS trial	30.1% (1066/3534) pre-delivery,8.5% (301/3534) post-delivery and 8.5% (302/3534) post-discharge
Shawa 2024 [32]	Cross-sectional	2015–2020	Lusaka	A tertiary care hospital: - in/outpatients (mixed)	NR
Yamba 2024 [62]	Cross-sectional	10/2020–01/2021	Lusaka, Copperbelt	Six primary care hospitals: - in/outpatients (pregnant women and children ≤5 years old)	above the WHO-recommended threshold of 30%
Yasmin 2024 [60]	Prospective, secondary analysis	2020–2022	Lusaka	Infants born to women enrolled in the A-PLUS trial	58/3562 (1.6%) post-delivery, 147/3562 (4.1%), post discharge

PPS, point prevalence study; WHO, World Health Organization; mixed∗, study included both adults and children.

### Quality assessment

The overall quality of the 17 cross section quantitative descriptive studies was generally good (≥75%) (see Supplementary Table A3). The main observed limitation was in determining the completeness of outcome data in many studies due to reliance on paper-based medical records. This often resulted in missing or unclear information regarding treatment duration, indication, and antibiotic rationale [32,48,50,54,55,51,62]. Whilst a few studies demonstrated data completeness, most lacked clear reporting on exclusions, the extent of missing data, or how such cases were handled [40,53,56,58,61]. Overall, data completeness remains a significant limitation affecting the quality of antimicrobial use evaluations in PPS studies.

The two before and after studies met all quality criteria for non-RCTs except that although the studies accounted for confounders it was limited by missing patient and clinical details [63,64].

### Antibiotic use prevalence

Among the studies that reported antibiotic use prevalence (Table II), prevalence was between 50.3% and 82.5% [35,61]. The highest reported prevalence was in a study conducted among adult in-patients in five primary hospitals [35]. Among the PPS studies, antibiotic use prevalence was between 59% and 79.8% [50,52]. Three studies reported ward specific prevalence. In one study, antibiotic use prevalence was highest in a neonatal intensive care unit (83.3%) and in the other two studies, prevalence was highest in the medical ward (80%) in one study [52] and in the female surgical ward (79%) in the other study [51].

Three studies assessed antimicrobial use in obstetric populations and were analyzed separately due to differences in study populations. Two studies from the multicounty A-Plus trial evaluated antibiotic use among pregnant women and neonates (0–42 days) across pre-delivery, post-delivery, and post-discharge periods. Among women, antibiotic use prevalence was 30.1% pre-delivery and 8.5% post-delivery, while neonatal use was 1.6% in-facility and 4.1% post-discharge [84,90]. Another study reported antibiotic use prevalence of 99.8% in women pre caesarean section surgery 89]. A further study evaluated trends in antibiotic consumption and AMR. However, whilst this study did not report overall antibiotic use prevalence, there was an observed association between increased third-generation cephalosporin use and higher resistance among *Enterobacterales* [54].

**Figure 3:**
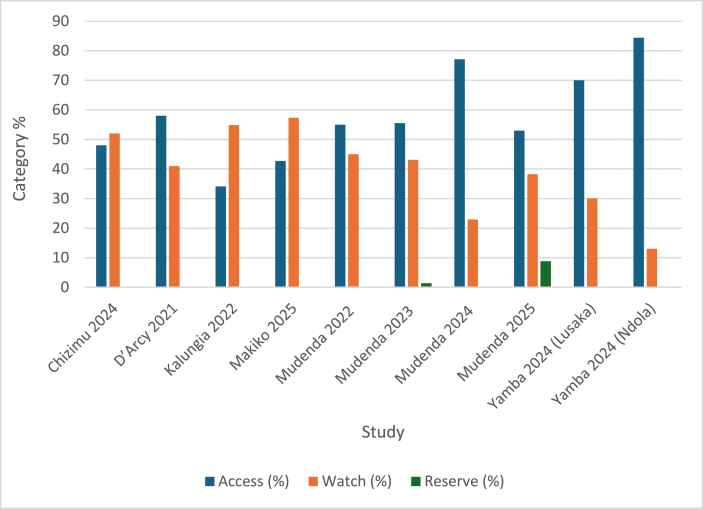
Antibiotic use by AWaRe categorization.

Based on the AWaRe categorization, most of the prescribed antibiotics were from the Access group of antibiotics, ranging from 34.2% - 84% of total antibiotic use [50,62]. However, there was appreciable prescribing of Watch antibiotics in the various studies, ranging from 13%-65.9%. Only two studies recorded the use of Reserve antibiotics of 1.4% and 8.8% of total antibiotic use [49,58] see, Figure 3.

**Figure 4:**
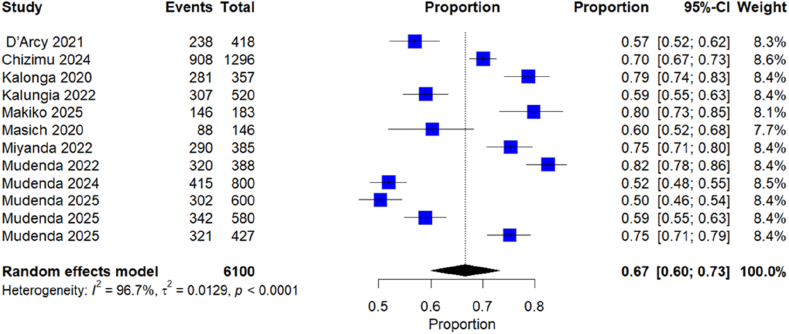
Forest plot of pooled prevalence of antibiotic use.

Overall, 12 studies provided comparable data and were included in a meta-analysis. The pooled estimate of antibiotic use prevalence was 67%, 95% CI (60–73) (see Figure 4), characterized by high heterogeneity (I^2^ = 96.7%, *P* < 0.000). A subgroup analysis was conducted based on study design, geographical location, health care setting or level and the study population; however, there was still high heterogeneity across the groups. The forest plots of the subgroup analysis are attached as supplementary data (Supplementary Figure A). There were no significant differences observed across subgroups, although subgroup analysis by location and setting (health care level) showed Lusaka (73%) and tertiary settings (72%) showed higher point estimates than the other groups.

Table III presents the predictors of antibiotic use. Across the five included studies, antibiotic use was most prevalent in children [48,53,51]. One study identified an association between gender and antibiotic use, linked to the administration of prophylactic antibiotics in gynaecological surgery [49]. In pediatrics, antibiotic use was associated with elevated white blood cell counts and the presence of systemic illnesses, particularly those affecting the respiratory system [53]. Taking account of the AWaRE classification, antibiotic prescriptions were associated with the route of administration and the ward. Access antibiotics more common in pediatric wards, and Watch antibiotics were more frequently prescribed in adult wards, with a high proportion of Watch antibiotics prescribed parenterally [52].

**Table III:** Predictors for antibiotic use

Study, year	Predictors for antibiotic use
Chizimu 2024 [48]	Antibiotic use was significantly higher among patients aged 15 years and below, and those treated in primary hospitals (*P* < 0.001), no significant difference in antibiotic use associated with ward type, human immune deficiency syndrome and malnutrition status, community acquired infection, hospital acquired infection, surgical and medical prophylaxis
D'Arcy 2021 [49]	Significant association between gender and antibiotic prescription across the Access, Watch and Reserve categories (X^2^ = 14.12, *P* < 0.001) with association attributed to indication for prophylaxis for obstetric or gynaecological surgery
Kalonga 2020 [53]	There was an association between age, white blood cell count status, and system of illness with antibiotic use (χ2 = 15.083, *P* = 0.001; χ2 = 6.571, *P* = 0.037 and χ2 = 27.717, *P* < 0.001) in paediatric patients.
Makiko 2024 [52]	Antibiotic prescribing by AWaRe classification was significantly associated with ward type and route of administration, with Access antibiotics prescribed more in paediatric wards 37/70 (52.9%) compared to adult wards 57/150 (38%). Watch antibiotics were predominantly used in adult wards 93/150 (62.0%) versus paediatric wards 33/70 (47.1%). Watch antibiotics were frequently prescribed parenterally 116/186 (62.4%) compared to the oral route 10/34 (29.4%), *P* = 0.0005.
Mudenda 2025 [51]	Antibiotics use prevalence was significantly higher in children aged 0–23 months 49/56 (87.5%), compared to other age groups (*P* = 0.001)

Table IV shows the top three most prescribed antibiotics across the included studies. In 8 out of 11 studies, antibiotics from the Watch group topped the list across all settings [35,[48], [49], [50],^52^,^55^,^51^,^58^], with ceftriaxone being the most prescribed in 7 of those 8 cases. The second most frequently prescribed antibiotics were from the Access group. Although there was some variation in the specific antibiotics, metronidazole was the most common prescribed antibiotic from the Access group.

**Table IV:** Three top prescribed antibiotics

Author, year	Healthcare level and population	Antibiotic	Frequency *n/N* (%)	AWaRE
Chizimu 2024 [48]	Six tertiary, five secondary and five primary care hospitals: - inpatients (mixed∗)	Ceftriaxone Benzylpenicillins Metronidazole	381/907 (42) 126/907 (14) 112/907 (12)	Watch Access Access
D'Arcy 2021 [49]	A tertiary and secondary care hospital: - inpatients (mixed)	Ceftriaxone Cloxacillin Benzylpenicillins	83/402 (20.6) 46/402 (11.4) 40/402 (10)	Watch Access Access
Kalungia 2022 [50]	10 primary and secondary care hospitals: - inpatients (mixed)	Ceftriaxone Cefotaxime Metronidazole	93/534 (36.1) 70/534 (13.1) 58/534 (10.9)	Watch Watch Access
Makiko 2024 [52]	Five primary care hospitals; - medical and surgical wards inpatients (mixed)	Ceftriaxone Metronidazole Benzylpenicillin	110/220 (50.0) 45/220 (20.5) 17/220 (7.7)	Watch Access Access
Masich 2020 [55]	A tertiary care hospital: - adult inpatient of medical wards	Cefotaxime Ceftriaxone Metronidazole	45/126 (35.7) 28/126 (22.2) 14/126 (11.1)	Watch Watch Access
Miyanda 2022 [56]	A primary care hospital: - adult inpatients of admission ward	Benzylpenicillin Cotrimoxazole Metronidazole	120/404 (29.7) 78/404 (19.3) 78/404 (19.3)	Access Access Access
Mudenda 2022 [35]	Five primary care hospitals adult in patients	Ceftriaxone Metronidazole sulfamethoxazole/trimethoprim<	65/320 (20.3) 57/320 (17.8) 52/320 (16.3)	Watch Access Access
Mudenda 2023 [58]	A tertiary care hospital: - in/outpatient (mixed)	Ceftriaxone Metronidazole Amoxicillin	118/443 (26.6) 100/443 (22.6) 46/443 (10.4)	Watch Access Access
Mudenda 2024 [40]	A secondary care hospital: - In/outpatients (mixed)	Amoxicillin Metronidazole Ciprofloxacin	135/578 (23.4) 99/578 (17.1) 46/578 (8.0)	Access Access Watch
Mudenda 2025 [51]	Five primary care hospitals: - inpatients (mixed)	Ceftriaxone Metronidazole Benzylpenicillin	231/631 (36.6) 135/631 (21.4) 49/631 (7.8)	Watch Access Access
Yamba 2024 [62]_Lusaka	Three primary care hospitals: - in/outpatients (pregnant women and children ≤5 years old)	Amoxicillin Metronidazole Ciprofloxacin	7272/32,205 (23) 5871/32,205 (18) 14,955/32,205 (15)	Access Access Watch
Yamba 2024 [62]_Ndola	Three primary care hospitals: - in/outpatients (pregnant women and children ≤5 years old)	Cefalexin Amoxicillin Metronidazole	7038/34,594 (20) 5834/34,594 (17) 5558/34,594 (16)	Access Access Access

AWaRe, Access, Watch and Reserve; ∗ study included both adults and children.

Five of the included studies, reported at least one indication for antibiotic use (Table V). Two studies reported whether the antibiotic use was for treatment or prophylaxis [49,56]. In the study by D'Arcy *et al.* (2021), 71.6% of antibiotics were indicated for treatment and 25.4% for prophylaxis [49]. In the study by Miyanda *et al.* (2022), 75.2% of antibiotics were indicated for treatment and only 4.8% were indicated for prophylaxis [56]. According to Table V, there was a trend of higher prevalence of antibiotics used in surgical and medical prophylaxis.

**Table V:** Reported indication for antibiotic use

Author, year	PR *N* (%)	TX *N* (%)	CAI *N* (%)	HAI *N* (%)	SAP *N* (%)	MP *N* (%)	Unknown *N* (%)	Others *N* (%)
Chizimu 2024 [48]	NR	NR	406/432 (94)	52/57 (91)	180/186 (97)	88/92 (96)	NR	NR
D'Arcy 2021 [49]	102/402 (25.4)	288/402 (71.6)	257/288 (89.2)	31/288 (10.8)	85/402 (21.1)	17/402 (4)	4/402 (1)	8/402 (2)
Kalungia 2022 [50]	NR	NR	261/534 (48.9)	NR	NR	11/534 (2.1)	NR	NR
Miyanda 2022 [56]	14/290 (4.8)	218/290 (75.2)	NR	NR	NR	NR	NR	NR
Mudenda 2025 [51]	NR	NR	148/150 (97)	18/19 (95)	61/61 (100)	80/81 (99)	NR	NR

PR, prophylaxis; TX, treatment; CAI, community-acquired infection; HAI, hospital-acquired infection; SAP, surgical antimicrobial prophylaxis; MP, medical practitioner; NR, not reported.

Two studies assessed the appropriateness of antibiotic use using different indicators (Table VI). Masich *et al.* (2020) evaluated antibiotic inappropriateness as a composite measure of correct dose, frequency, route, duration, and spectrum of coverage for each indication based on the Infectious Diseases Society of America (IDSA) guidelines, WHO guidelines, and local infectious diseases clinical expertise [55], reporting an overall inappropriateness of 67%, with generally varying levels of inappropriateness across assessed indicators [55]. However, Mudenda *et al.* (2023) primarily assessed antibiotic appropriateness by evaluating adherence to the 2020 Zambian Standard Treatment Guidelines (STGs), and reported appropriateness of 98.9% for indication, 99.5% for dose, 99.7% for frequency, and 96.9% for duration [58].

**Table VI:** Antibiotic use appropriateness

Author, year	Number of patients	Prevalence %	Indication *N* (%)	Drug choice *N* (%)	Dose *N* (%)	Dosing frequency *N* (%)	Duration *N* (%)	Route of administration *N* (%)	Redundancy *N* (%)	Overall appropriateness *N* (%)
Masich 2020 [55] a	88	60	8 (9.1)	21 (24)	26 (29.5)	26 (29.5)	2 (2.3)	15 (17)	11 (12.5)	59 (67)
Mudenda 2023 [58] a	384	NR	380 (98.9)	NR	382 (99.5)	383 (99.7)	372 (96.9	NR	NR	NR

a Masich reported proportion of inappropriate prescribing while Mudenda reported antibiotic appropriateness.

Overall, there was low reporting of quality indicators for antibiotic use with only nine of the 19 included studies reporting at least one quality indicator for antibiotic use. The most reported quality indicators were compliance with national STGs alongside documentation of the indication, which was reported in eight and five studies, respectively, see Table VII. Compliance with STGs ranged from 27% to 58.1% [35,[48], [49], [50],^52^,^56^,^51^,^65^], while documentation of the indication for prescribing varied from 15.7% to 80% [49,50,52]. Microbiology testing was infrequently reported, with only three studies including this indicator, showing rates of 0%, 3% and 32% [50,52,55]. Only one study reported on surgical antibiotic prophylaxis (SAP), in which 96.5% of cases exceeded the recommended 24-h duration [49].

**Table VII:** Quality indicators for antibiotic use

Author, year	Indication for antibiotic use (%)	Start/review dates indicated (%)	Redundant prescriptio*N* (%)	Intravenous antibiotic (%)	Oral antibiotic (%)	Microbiology test (%)	Targeted treatment (%)	Empirical treatment (%)	Guideline compliance (%)	SP >24hrs
Chizimu 2024 [48]	NR	NR	NR	NR	NR	NR	NR	NR	480/908 (53)	NR
D'Arcy 2021 [49]	321/402 (80)	82/402 (20)	NR	NR	NR	NR	NR	NR	195/402 (55)	83/402 (96.5)
Kalungia 2022 [50]	84/534 (15.7)	171/534 (32)	NR	NR	NR	16/534 (3)	16/534 (3)	518/534 (97)	144/534 (27)	NR
Makiko 2025 [52]	147/220 (66.8)	11/220 (5)	NR	NR	NR	0	220/220 (100)	0	81/220 (36.8)	NR
Masich 2020 [55]	NR	NR	11/88 (12.5)	NR	NR	28/88 (32)	NR	NR	NR	NR
Miyanda 2022 [56]	NR	NR	NR	NR	NR	NR	NR	NR	148/290 (51.1)	NR
Mudenda 2022 [35]	NR	NR	NR	NR	NR	NR	NR	NR	186/388 (58.1)	NR
Mudenda 2025 [51]	NR	NR	NR	NR	NR	NR	3/342 (0.9)	NR	124/342 (36)	NR
Mudenda 2025 [65]	NR	NR	NR	NR	NR	NR	NR	NR	137/427 (44)	NR

### Impact of antimicrobial stewardship

Two studies evaluated the impact of ASPs. Kalungia *et al.* (2024) employed a hub-and-spoke model to establish ASPs in 10 Zambian hospitals that previously lacked such programs (Table VIII). After 12 months, there was a significant improvement in the adoption of ASP core elements across these hospitals (*P* = 0.001, 95% CI: −17.8 to −5.42) [64]. Overall antibiotic use decreased from 50.1% at baseline to 44.3% post-intervention. Access antibiotic use showed minimal change (50.1% vs. 50.9%), while Watch antibiotic use also remained largely unchanged (48.8% vs. 48.6%), with reductions observed in only 50% of the participating hospitals (5/10) [64]. Mudenda *et al.* (2025) employed a multifaceted ASP program involving the establishment or strengthening of ASP programs in three hospitals. The ASP showed an early positive impact on antibiotic use and prescribing patterns among the three Zambian hospitals. There was a 10% decrease in overall antibiotic use prevalence and a 10% reduction in ceftriaxone prescribing [65].

**Table VIII:** Impact of antimicrobial stewardship

Author, year	Intervention employed and adoption	Antibiotic utilisation	Frequently prescribed AWaRe antibiotics
Kalungia 2024 [64] Pre vs Post sample: 1477 vs 1400, across 10 across tertiary and secondary level hospitals	A multidisciplinary team from the hub hospital provided mentorship and ASP training, while spoke hospitals implemented action plans using plan-to-study-act cycles, monitored at 6 and 12 months. Post-intervention, over 80% of ASP core elements were partially implemented across all hospitals, with a significant increase in adoption compared with baseline (mean difference = 11.6 ± 2.94; *P* = 0.001; 95% CI: −17.8 to −5.42)	From pre- to post-intervention, overall AUP decreased by 5.8% across the 10 spoke hospitals. By ward type, AUP declined by 4.8% (*P* = 0.554) in adult medical wards. 5.7% (*P* = 0.522) in adult surgical wards (), and 18.3% (*P* = 0.412) in the adult ICU while a 1.6% (*P* = 0.866) was observed in paediatric medical wards	Across all hospitals, the mean AUP for Access and Watch antibiotics remained almost unchanged pre to post intervention. No Reserve antibiotics were recorded pre-int, post-int, two hospitals reported 2% and 3% AUP for Reserve antibiotics
Mudenda 2025 [65] Pre vs post sample:172 vs 265 across three tertiary hospitals	A 3-year ASP demonstration project was implemented in three tertiary hospitals to optimize antibiotic use for urinary tract and bloodstream infections. The intervention combined staff training, multidisciplinary ASP rounds, real-time prescriber feedback, and promotion of adherence to national treatment guidelines through assessments and awareness activities.	A 10% reduction of antibiotic use from pre to post intervention) Compliance with national STGs was significantly associated with survey year (*P* < 0.001), with better adherence observed in 2023 than in 2022. Overall proportion of compliant prescriptions showed only a marginal change, increasing from 42% in 2022 to 45% in 2023.	Pre-intervention, Access and Watch antibiotics each accounted for 50% of prescriptions; post-intervention, Access use declined to 43% while Watch increased to 57%. Ceftriaxone prescribing decreased from an average of 48% in 2022 to 38% in 2023.

ASP, antimicrobial stewardship; AUP, antibiotic use prevalence; ICU, intensive care units.

## Discussion

To our knowledge, this is the first systematic review of hospital antibiotic use in Zambia with significant observed increase in publications since 2020 reflecting growing efforts to generate evidence to support nation action on AMR in line with the goals of the NAP [38]. The review found a high prevalence of antibiotic use, ranging from 50.3% to 82.5%, which is similar to previous sub-Sahara Africa reviews reporting hospital prevalence of 37.7%–80.1% [66], and up to 97.8% [18]. A more recent review across Africa reported antibiotic use prevalence of 27.8%–83.5% [67], which is notably higher than Europe (30.5%) [68], the USA (49.9%) [69] and in the Global-PPS study where African countries showed the highest antibiotic use prevalence of 50% (27.8–74.7%) [27]. The pooled estimate from the meta-analysis was 67%. These findings are concerning, particularly given the established association between high antibiotic use and the development of AMR [7,70]. However, this may reflect higher rates of infectious disease in Africa than seen in Europe and the North Americas along with higher rates of AMR in Africa [6,18]. Along with this, concerns with issues such as poor infection prevention and control among African hospitals compared with those in high-income countries [66,67,71]. Antibiotic use prevalence was highest in pediatric ICU, medical, and female surgical wards, especially in children, surgical prophylaxis and respiratory infections [49,50,52,51]. This is similar to identified predictors of antibiotic use, which show a greater likelihood of use in children and prophylaxis in gynecological surgery [48,49,53,51]. Whilst Access antibiotics were most prescribed overall; Watch antibiotics, particularly Ceftriaxone was the most prescribed antibiotic reflecting and reaffirming a broader trend of common cephalosporins prescribing across hospitals in LMICs [67,72].

Only two studies assessed the appropriateness of antibiotic prescribing in hospital settings, with one reporting a high level of inappropriate use while the other found good adherence to current STGs [55,58]. Further research is needed to strengthen the evidence base in this area, including instigating pertinent quality indicators among hospitals across Zambia based on the AWaRe system and guidance, subsequently monitoring adherence rates [18,25]. The ongoing refinement of quality indicators based on the AWaRe system and guidance should further help here [73]. The increasing use of quality indicators should help reduce the high levels of inappropriate antibiotic use, with an estimation of less than 70% compliance to prescribing standards [27,66]. The findings highlight challenges in optimizing antibiotic use in Zambian hospitals and can guide research and interventions to support effective ASPs. Similarly to appropriateness, few studies report quality indicators for antibiotic use, and generally reveal low compliance [35,[48], [49], [50],^52^,[55], [56], [57], [58],^65^]. Guideline adherence was the most reported quality indicator but remained suboptimal [[48], [49], [50],^56^,^51^]. This was despite evidence showing a 35% mortality reduction with guideline-based therapy [66,74]. This gap is likely influenced by clinician prescribing habits, lack of awareness or access to updated guidelines, alongside concerns with national antibiotic guidelines among LMICs, and systemic barriers within healthcare settings, which include limited diagnostic capacity, insufficient laboratory support, and understaffed healthcare facilities [18,74,75]. Obtaining microbiological culture and sensitivity testing prior to indicating antibiotics is essential for targeted therapy and reducing AMR [67,75], however, findings show microbiology testing in Zambian hospitals remain limited. This must be addressed going forward. Furthermore, SAP was typically more than 24 h, which is contrary to best practice recommendations adding to AMR [18,66,76,77]. This mirrors patterns across sub-Saharan Africa, where targeted therapy is as low as 10% due to infrastructure and cost barriers, and approximately 84% of SAP courses are prolonged [66,67]. Similar patterns are observed in neighbouring countries to Zambia. This includes Botswana where prolonged SAP was seen in 90–100% of cases with low culture utilization; in Tanzania with estimates that 50% of SAP exceeds 3 days; Kenya where there have been reports of prolonged SAP in 76.9% of cases; and in South Africa, where 73.2% of adults and 66.7% of pediatric surgical patients receive SAP beyond 24 h [18,66,78]. Such situations reflect overall suboptimal guideline compliance in sub-Saharan Africa, with estimates of 48–49% [66]. Compliance to guidelines among the limited number of studies assessing this in our review was lower than Tanzania (84%) and South Africa (90.2%) [66,79]. However comparable to Uganda (30–67%), Zimbabwe (57.7%), and Kenya (45.8%) [49,50,79]. Regional similarities are primarily due to a shared high burden of infectious diseases and limited diagnostic resources [66,67].

Excessive SAP, combined with insufficient monitoring and involving broad-spectrum Watch agents, creates a significant selective pressure that drives AMR and healthcare cost [13,66,76]. Encouragingly, two studies showed that ASPs improved the implementation of stewardship activities and contributed to a reduction in overall antibiotic use. However, the continued high use of Watch antibiotics, particularly ceftriaxone, indicates that changes in prescribing behaviour remain limited [64,65]. These findings suggest that while ASPs can establish an important foundation, sustained and more intensive interventions are needed to achieve meaningful and lasting improvements in antibiotic use in hospitals across Zambia [64,65]. Earlier systematic reviews and meta-analysis of ASP interventions in Africa affirms that these interventions are likely to be effective, providing reassurance to key stakeholders across Zambia [23,24,79]. Their findings broadly support the notion that ASP interventions lead to increased compliance with targeted practices and a reduction in antibiotic utilization and cost. Having said this, more studies that assess the impact of ASP in Zambia are crucial to further build the evidence base in Zambia. Emphasis should be on possible ways to address underlying structural and systemic challenges in improving antibiotic use in hospitals in Zambia and their impact.

### Limitations and strengths

We are aware of a number of limitations with our review. Firstly, there was no registered protocol, which may limit transparency and introduce the potential for selective reporting. This was mitigated though by using predefined inclusion criteria, a comprehensive and systematic search strategy, and transparent reporting of all methods and outcomes.

Additionally, most of the available data originate from studies conducted in Lusaka, which may limit the generalizability of the findings to other regions. Another limitation to note is that grey literature and unpublished studies were not included, which may have led to an overrepresentation of studies with positive or significant findings. Regarding study design, the predominance of cross-sectional and point prevalence studies restricts causal interpretation. These methods capture antibiotic use at a single time point and may not reflect seasonal variation, duration of therapy, or changes over time. Data quality limitations such as incomplete records, missing documentation of indication and stop/review dates, limited linkage to microbiological results, and variability in survey implementation and ward coverage, may also have introduced measurement bias and influenced the observed antibiotic utilization trends. A lack of standardized outcome reporting across studies further complicates comparisons and synthesis. These limitations underscore the need for broader, methodologically rigorous research across diverse settings in Zambia.

The strength of this review lies in that to the best of our knowledge; it is the first comprehensive systematic review of antibiotic use among hospitals in Zambia. Consequently, providing valuable insights to all key stakeholder groups in Zambia by examining multiple domains influencing antibiotic use practices. These include utilization patterns, AWaRe categorization, appropriateness, quality indicators, and stewardship across hospital setting. The findings serve as a baseline for future interventions. It also highlights critical gaps in data and practice. Overall, we believe this review adds important context to the national effort to address AMR.

In conclusion, antibiotic utilization in Zambian is characterised by a high prevalence across all levels of care, frequently exceeding WHO threshold of 30%. The prescribing patterns are primarily driven by limited diagnostic infrastructure and sub-optimal adherence to national guidelines, though multifaceted stewardship initiatives have shown early success in optimizing prescribing quality. It is important that further ASPs are undertaken among hospitals across Zambia to address current concerns with antibiotic use including appreciable prescribing of Watch antibiotics such as ceftriaxone.

## Policy recommendations

To combat inappropriate antibiotic use and AMR in Zambia, the health authorities should mainstream ASPs across all hospital levels, ensuring formal accountability and dedicated funding. This may require universities in Zambia to re-assess their current curricula to ensure this adequately covers all key aspects of the AWaRe system as well as how to effectively implement ASPs. Additionally, health facilities must integrate WHO AWaRe monitoring and guidance into national guidelines and local protocols to prioritize Access-group antibiotics and achieve the UN-GA target of 70% utilization. To address limited microbiological diagnostic capacity, significant investment in laboratory infrastructure is also required to reduce the current 97% empiric prescribing rate.

Actionable steps among hospitals across Zambia include expanding biannual PPS audits and implementing automated antibiotic stop orders through standardised antibiotic prescription charts to curb prolonged surgical prophylaxis. Furthermore, authorities together with Universities should assess and implement agreed quality indicators based on the AWaRe system and guidance and subsequently monitor their use. This will necessitate increasing the specialized workforce and empowering health workers through formalized stewardship roles and continuous professional development regarding ASPs. National STGs must also be regularly updated based on AWaRe guidance using local antibiogram data, with adherence subsequently monitored through ASPs. These interventions are needed to enhance future appropriate antibiotic use among hospitals in Zambia to reduce AMR.

## CRediT authorship contribution statement

**Linda Siachalinga:** Writing – review & editing, Writing – original draft, Visualization, Software, Project administration, Methodology, Investigation, Formal analysis, Data curation, Conceptualization. **Newton Nyirenda:** Writing – review & editing, Writing – original draft, Visualization, Validation, Investigation, Data curation. **Mwiza Hachalinga:** Writing – review & editing, Writing – original draft, Visualization, Validation, Investigation, Data curation. **Ronald Kampamba Mutati:** Writing – review & editing, Writing – original draft, Validation. **Webrod Mufwambi:** Writing – review & editing, Writing – original draft, Validation. **Aubrey Chichonyi Kalungia:** Writing – review & editing, Writing – original draft, Validation, Supervision, Resources, Project administration, Data curation. **Brian Godman:** Writing – review & editing, Writing – original draft, Validation, Supervision, Resources, Project administration, Data curation. **Hansoo Kim:** Writing – review & editing, Writing – original draft, Validation, Supervision, Resources, Project administration, Data curation.

## Ethics statement

Not required.

## Funding sources

This study was funded by Griffith University.

## Conflict of interest statement

None declared.
