# Supplementary material for: Antibiotic utilization in outpatient and inpatient hospitals in Zambia: a systematic review, key findings and public health implications
Source: Infect Prev Pract. 2026 Apr 23;8(2):100547. doi: 10.1016/j.infpip.2026.100547 (PMC13223719; doi:10.1016/j.infpip.2026.100547)

Figure. A1 subgroup analysis by study design

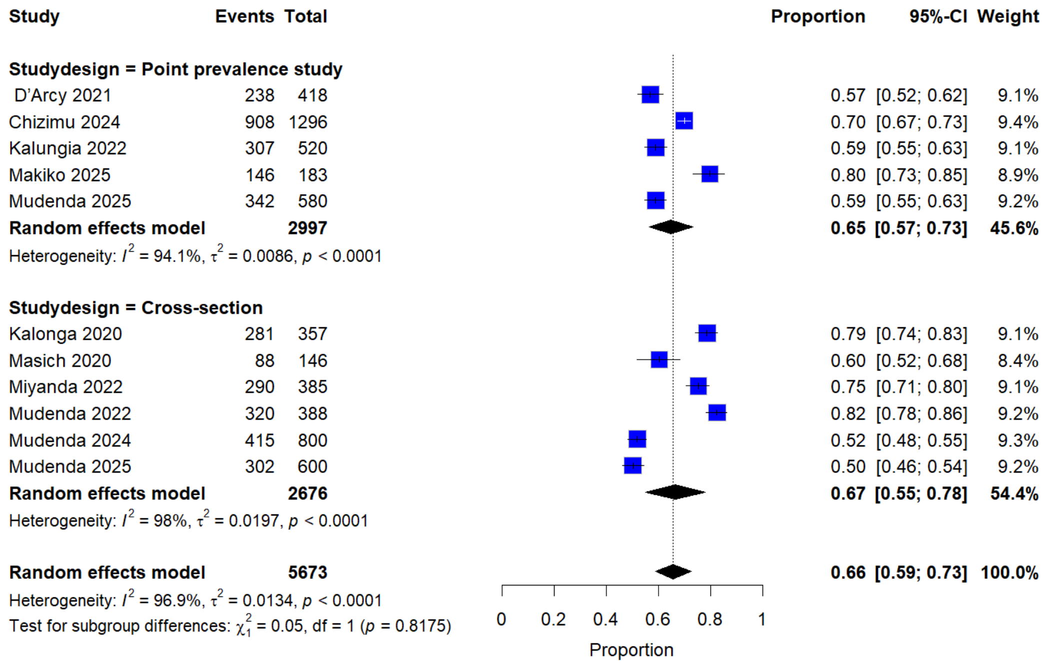

Figure. A2 subgroup analysis by setting

Study	Events	Total	Proportion	95%-CI	Weight
**Setting = Mixed**					
D'Arcy 2021	238	418	0.57	[0.52; 0.62]	9.1%
Chizimu 2024	908	1296	0.70	[0.67; 0.73]	9.4%
Kalungia 2022	307	520	0.59	[0.55; 0.63]	9.2%
**Random effects model**		**2234**	**0.62**	**[0.54; 0.70]**	**27.6%**
Heterogeneity: $I^2 = 94.2\%$, $\tau^2 = 0.0048$, $p < 0.0001$					
**Setting = Tertiary**					
Kalonga 2020	281	357	0.79	[0.74; 0.83]	9.2%
Masich 2020	88	146	0.60	[0.52; 0.68]	8.4%
Mudenda 2025	321	427	0.75	[0.71; 0.79]	9.2%
**Random effects model**		**930**	**0.72**	**[0.61; 0.82]**	**26.7%**
Heterogeneity: $I^2 = 87.6\%$, $\tau^2 = 0.0080$, $p = 0.0003$					
**Setting = Primary**					
Makiko 2025	146	183	0.80	[0.73; 0.85]	8.9%
Miyanda 2022	290	385	0.75	[0.71; 0.80]	9.2%
Mudenda 2022	320	388	0.82	[0.78; 0.86]	9.2%
Mudenda 2025	302	600	0.50	[0.46; 0.54]	9.2%
Mudenda 2025	342	580	0.59	[0.55; 0.63]	9.2%
**Random effects model**		**2136**	**0.69**	**[0.57; 0.82]**	**45.7%**
Heterogeneity: $I^2 = 97.7\%$, $\tau^2 = 0.0192$, $p < 0.0001$					
**Random effects model**		**5300**	**0.68**	**[0.61; 0.75]**	**100.0%**
Heterogeneity: $I^2 = 96.1\%$, $\tau^2 = 0.0118$, $p < 0.0001$					
Test for subgroup differences: $\chi^2_2 = 2.25$, df = 2 ($p = 0.3242$)					

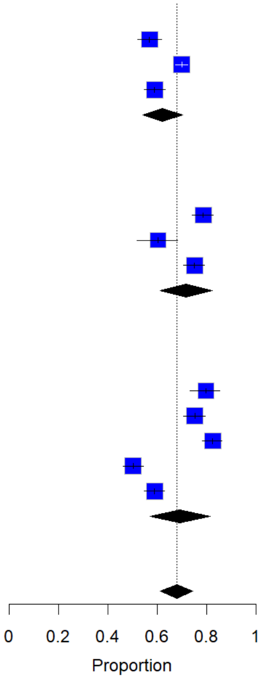

Figure. A3 subgroup analysis by population

Study	Events	Total	Proportion	95%-CI	Weight
**Population = Mixed**					
D'Arcy 2021	238	418	0.57	[0.52; 0.62]	9.1%
Chizimu 2024	908	1296	0.70	[0.67; 0.73]	9.4%
Kalungia 2022	307	520	0.59	[0.55; 0.63]	9.2%
Makiko 2025	146	183	0.80	[0.73; 0.85]	8.9%
Mudenda 2024	415	800	0.52	[0.48; 0.55]	9.3%
Mudenda 2025	302	600	0.50	[0.46; 0.54]	9.2%
Mudenda 2025	342	580	0.59	[0.55; 0.63]	9.2%
**Random effects model**		**4397**	**0.61**	**[0.53; 0.69]**	**64.1%**
Heterogeneity: $I^2 = 96\%$, $\tau^2 = 0.0102$, $p < 0.0001$					
**Population = Adults**					
Masich 2020	88	146	0.60	[0.52; 0.68]	8.4%
Miyanda 2022	290	385	0.75	[0.71; 0.80]	9.1%
Mudenda 2022	320	388	0.82	[0.78; 0.86]	9.2%
Mudenda 2025	321	427	0.75	[0.71; 0.79]	9.2%
**Random effects model**		**1346**	**0.74**	**[0.65; 0.82]**	**35.9%**
Heterogeneity: $I^2 = 88.6\%$, $\tau^2 = 0.0070$, $p < 0.0001$					
**Random effects model**		**5743**	**0.65**	**[0.59; 0.72]**	**100.0%**
Heterogeneity: $I^2 = 96.7\%$, $\tau^2 = 0.0126$, $p < 0.0001$					
Test for subgroup differences: $\chi^2_1 = 4.80$, df = 1 ($p = 0.0284$)					

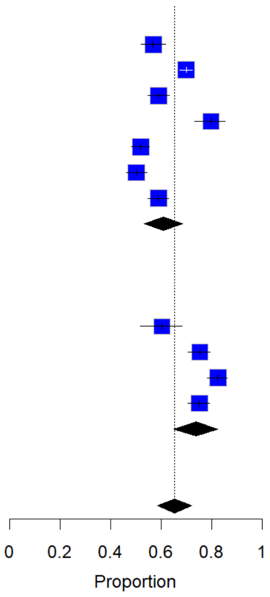

Figure. A4 subgroup analysis by location

Study	Events	Total	Proportion	95%-CI	Weight
**Location = ≥ 2 provinces**					
D'Arcy 2021	238	418	0.57	[0.52; 0.62]	10.0%
Mudenda 2025	321	427	0.75	[0.71; 0.79]	10.1%
**Random effects model**		**845**	**0.66**	**[0.48; 0.84]**	**20.1%**
Heterogeneity: $I^2 = 96.9\%$, $\tau^2 = 0.0161$, $p < 0.0001$					
**Location = Across Zambia**					
Chizimu 2024	908	1296	0.70	[0.67; 0.73]	10.4%
Kalungia 2022	307	520	0.59	[0.55; 0.63]	10.1%
**Random effects model**		**1816**	**0.65**	**[0.54; 0.75]**	**20.6%**
Heterogeneity: $I^2 = 94.8\%$, $\tau^2 = 0.0058$, $p < 0.0001$					
**Location = Lusaka**					
Kalonga 2020	281	357	0.79	[0.74; 0.83]	10.1%
Makiko 2025	146	183	0.80	[0.73; 0.85]	9.7%
Masich 2020	88	146	0.60	[0.52; 0.68]	9.0%
Miyanda 2022	290	385	0.75	[0.71; 0.80]	10.1%
Mudenda 2022	320	388	0.82	[0.78; 0.86]	10.2%
Mudenda 2025	342	580	0.59	[0.55; 0.63]	10.2%
**Random effects model**		**2039**	**0.73**	**[0.65; 0.81]**	**59.3%**
Heterogeneity: $I^2 = 94.7\%$, $\tau^2 = 0.0098$, $p < 0.0001$					
**Random effects model**		**4700**	**0.70**	**[0.64; 0.76]**	**100.0%**
Heterogeneity: $I^2 = 94.7\%$, $\tau^2 = 0.0092$, $p < 0.0001$					
Test for subgroup differences: $\chi^2_2 = 1.51$, df = 2 ($p = 0.4702$)					

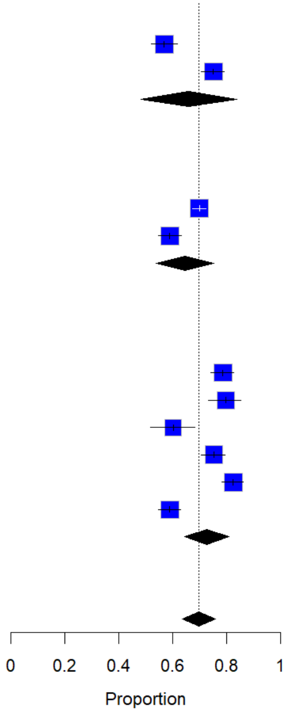

Supplement: Multimedia component 1 [file mmc1.pdf]
