# Supplementary material for: Antibiotic utilization in outpatient and inpatient hospitals in Zambia: a systematic review, key findings and public health implications
Source: Infect Prev Pract. 2026 Apr 23;8(2):100547. doi: 10.1016/j.infpip.2026.100547 (PMC13223719; doi:10.1016/j.infpip.2026.100547)
Supplement: Multimedia component 3 [file mmc3.docx]

**Supplementary Table A2. Search Strategy**

**Table 1. PubMed**

| Search number | Query |
| --- | --- |
| 12 | (((((((((antibiotic[MeSH Terms]) OR (antimicrobial[MeSH Terms])) OR (anti-bacteria)) OR (anti-bacterial agents)) OR (antibiotic prophylaxis)) AND ((((((((use) OR (usage)) OR (utilization)) OR (consumption)) OR (prescribing)) OR (prescription)) OR (dispensing)) OR (practice))) OR ((((((antibiotic[MeSH Terms]) OR (antimicrobial[MeSH Terms])) OR (anti-bacteria)) OR (anti-bacterial agents)) OR (antibiotic prophylaxis)) AND ((((((((((intervention) OR (programme)) OR (stewardship)) OR (antimicrobial stewardship)) ) OR (quality improvement)) OR (guidelines)) OR (adherence)) OR (policies)) OR (education)))) AND ((((hospital) OR (inpatient)) OR (outpatient)) OR (ambulatory))) AND (Zambia) AND (2000:2025[pdat])) NOT (((Human Immune deficiency virus) OR (Tuberculosis)) OR (TB)) |
| 11 | ((Human Immune deficiency virus) OR (Tuberculosis)) OR (TB) |
| 10 | ((((((((antibiotic[MeSH Terms]) OR (antimicrobial[MeSH Terms])) OR (anti-bacteria)) OR (anti-bacterial agents)) OR (antibiotic prophylaxis)) AND ((((((((use) OR (usage)) OR (utilization)) OR (consumption)) OR (prescribing)) OR (prescription)) OR (dispensing)) OR (practice))) OR ((((((antibiotic[MeSH Terms]) OR (antimicrobial[MeSH Terms])) OR (anti-bacteria)) OR (anti-bacterial agents)) OR (antibiotic prophylaxis)) AND ((((((((((intervention) OR (programme)) OR (stewardship)) OR (antimicrobial stewardship)) ) OR (quality improvement)) OR (guidelines)) OR (adherence)) OR (policies)) OR (education)))) AND ((((hospital) OR (inpatient)) OR (outpatient)) OR (ambulatory))) AND (Zambia) |
| 9 | ((((((((antibiotic[MeSH Terms]) OR (antimicrobial[MeSH Terms])) OR (anti-bacteria)) OR (anti-bacterial agents)) OR (antibiotic prophylaxis)) AND ((((((((use) OR (usage)) OR (utilization)) OR (consumption)) OR (prescribing)) OR (prescription)) OR (dispensing)) OR (practice))) OR ((((((antibiotic[MeSH Terms]) OR (antimicrobial[MeSH Terms])) OR (anti-bacteria)) OR (anti-bacterial agents)) OR (antibiotic prophylaxis)) AND ((((((((((intervention) OR (programme)) OR (stewardship)) OR (antimicrobial stewardship)) ) OR (quality improvement)) OR (guidelines)) OR (adherence)) OR (policies)) OR (education)))) AND ((((hospital) OR (inpatient)) OR (outpatient)) OR (ambulatory))) AND (Zambia) |
| 8 | ((((((antibiotic[MeSH Terms]) OR (antimicrobial[MeSH Terms])) OR (anti-bacteria)) OR (anti-bacterial agents)) OR (antibiotic prophylaxis)) AND ((((((((use) OR (usage)) OR (utilization)) OR (consumption)) OR (prescribing)) OR (prescription)) OR (dispensing)) OR (practice))) OR ((((((antibiotic[MeSH Terms]) OR (antimicrobial[MeSH Terms])) OR (anti-bacteria)) OR (anti-bacterial agents)) OR (antibiotic prophylaxis)) AND ((((((((((intervention) OR (programme)) OR (stewardship)) OR (antimicrobial stewardship)) ) OR (quality improvement)) OR (guidelines)) OR (adherence)) OR (policies)) OR (education))) |
| 7 | (((((antibiotic[MeSH Terms]) OR (antimicrobial[MeSH Terms])) OR (anti-bacteria)) OR (anti-bacterial agents)) OR (antibiotic prophylaxis)) AND ((((((((((intervention) OR (programme)) OR (stewardship)) OR (antimicrobial stewardship)) ) OR (quality improvement)) OR (guidelines)) OR (adherence)) OR (policies)) OR (education)) |
| 6 | (((((antibiotic[MeSH Terms]) OR (antimicrobial[MeSH Terms])) OR (anti-bacteria)) OR (anti-bacterial agents)) OR (antibiotic prophylaxis)) AND ((((((((use) OR (usage)) OR (utilization)) OR (consumption)) OR (prescribing)) OR (prescription)) OR (dispensing)) OR (practice)) |
| 5 | Zambia |
| 4 | (((hospital) OR (inpatient)) OR (outpatient)) OR (ambulatory) |
| 3 | (((((((((intervention) OR (programme)) OR (stewardship)) OR (antimicrobial stewardship)) ) OR (quality improvement)) OR (guidelines)) OR (adherence)) OR (policies)) OR (education) |
| 2 | (((((((use) OR (usage)) OR (utilization)) OR (consumption)) OR (prescribing)) OR (prescription)) OR (dispensing)) OR (practice) |
| 1 | ((((antibiotic[MeSH Terms]) OR (antimicrobial[MeSH Terms])) OR (anti-bacteria)) OR (anti-bacterial agents)) OR (antibiotic prophylaxis) |

**Table 2. Embase**

| No. | Query |
| --- | --- |
| #11 | ('antibiotic'/exp OR 'antibiotic' OR 'antimicrobial'/exp OR 'antimicrobial' OR 'anti-bacterial agent'/exp OR 'anti-bacterial agent' OR 'antibiotic prophylaxis'/exp OR 'antibiotic prophylaxis') AND ('hospital'/exp OR 'inpatient'/exp OR 'outpatient'/exp OR 'ambulatory') AND 'zambia'/exp AND (('use' OR 'usage' OR 'utilization'/exp OR 'consumption'/exp OR 'prescribing' OR 'prescription'/exp) OR ('dispensing' OR 'practice'/exp OR 'intervention'/exp OR 'programme' OR 'stewardship'/exp) OR ('antimicrobial stewardship'/exp OR 'quality improvement'/exp OR 'guidelines'/exp OR 'adherence'/exp OR 'policies' OR 'education'/exp)) AND [2000-2025]/py |
| #10 | ('antibiotic'/exp OR 'antibiotic' OR 'antimicrobial'/exp OR 'antimicrobial' OR 'anti-bacterial agent'/exp OR 'anti-bacterial agent' OR 'antibiotic prophylaxis'/exp OR 'antibiotic prophylaxis') AND ('hospital'/exp OR 'inpatient'/exp OR 'outpatient'/exp OR 'ambulatory') AND 'zambia'/exp AND (('use' OR 'usage' OR 'utilization'/exp OR 'consumption'/exp OR 'prescribing' OR 'prescription'/exp) OR ('dispensing' OR 'practice'/exp OR 'intervention'/exp OR 'programme' OR 'stewardship'/exp) OR ('antimicrobial stewardship'/exp OR 'quality improvement'/exp OR 'guidelines'/exp OR 'adherence'/exp OR 'policies' OR 'education'/exp)) |
| #9 | ('antibiotic'/exp OR 'antibiotic' OR 'antimicrobial'/exp OR 'antimicrobial' OR 'anti-bacterial agent'/exp OR 'anti-bacterial agent' OR 'antibiotic prophylaxis'/exp OR 'antibiotic prophylaxis') AND ('hospital'/exp OR 'inpatient'/exp OR 'outpatient'/exp OR 'ambulatory') AND 'zambia'/exp AND (('use' OR 'usage' OR 'utilization'/exp OR 'consumption'/exp OR 'prescribing' OR 'prescription'/exp) OR ('dispensing' OR 'practice'/exp OR 'intervention'/exp OR 'programme' OR 'stewardship'/exp) OR ('antimicrobial stewardship'/exp OR 'quality improvement'/exp OR 'guidelines'/exp OR 'adherence'/exp OR 'policies' OR 'education'/exp)) |
| #8 | ('antibiotic'/exp OR 'antibiotic' OR 'antimicrobial'/exp OR 'antimicrobial' OR 'anti-bacterial agent'/exp OR 'anti-bacterial agent' OR 'antibiotic prophylaxis'/exp OR 'antibiotic prophylaxis') AND ('hospital'/exp OR 'inpatient'/exp OR 'outpatient'/exp OR 'ambulatory') AND 'zambia'/exp AND (('use' OR 'usage' OR 'utilization'/exp OR 'consumption'/exp OR 'prescribing' OR 'prescription'/exp) OR ('dispensing' OR 'practice'/exp OR 'intervention'/exp OR 'programme' OR 'stewardship'/exp) OR ('antimicrobial stewardship'/exp OR 'quality improvement'/exp OR 'guidelines'/exp OR 'adherence'/exp OR 'policies' OR 'education'/exp)) |
| #7 | ('use' OR 'usage' OR 'utilization'/exp OR 'consumption'/exp OR 'prescribing' OR 'prescription'/exp) OR ('dispensing' OR 'practice'/exp OR 'intervention'/exp OR 'programme' OR 'stewardship'/exp) OR ('antimicrobial stewardship'/exp OR 'quality improvement'/exp OR 'guidelines'/exp OR 'adherence'/exp OR 'policies' OR 'education'/exp) |
| #6 | 'zambia'/exp |
| #5 | 'hospital'/exp OR 'inpatient'/exp OR 'outpatient'/exp OR 'ambulatory' |
| #4 | 'antimicrobial stewardship'/exp OR 'quality improvement'/exp OR 'guidelines'/exp OR 'adherence'/exp OR 'policies' OR 'education'/exp |
| #3 | 'dispensing' OR 'practice'/exp OR 'intervention'/exp OR 'programme' OR 'stewardship'/exp |
| #2 | 'use' OR 'usage' OR 'utilization'/exp OR 'consumption'/exp OR 'prescribing' OR 'prescription'/exp |
| #1 | 'antibiotic'/exp OR 'antibiotic' OR 'antimicrobial'/exp OR 'antimicrobial' OR 'anti-bacterial agent'/exp OR 'anti-bacterial agent' OR 'antibiotic prophylaxis'/exp OR 'antibiotic prophylaxis' |
