# Supplementary material for: Antibiotic utilization in outpatient and inpatient hospitals in Zambia: a systematic review, key findings and public health implications
Source: Infect Prev Pract. 2026 Apr 23;8(2):100547. doi: 10.1016/j.infpip.2026.100547 (PMC13223719; doi:10.1016/j.infpip.2026.100547)
Supplement: Multimedia component 4 [file mmc4.pdf]

# Supplementary Table A3 – Mixed Methods Appraisal Tool Results

## Quantitative Non.RCTs studies

| Author/Year | Title | MMAT score (% | Quality | 1.1 | 1.2 | 3.1 | 3.2 | 3.3 | 3.4 | 3.5 |
|---|---|---|---|---|---|---|---|---|---|---|
| Kalungia 2024 | Impact of a hub-and-spoke approach to hospital antimicrobial stewardship programmes on antibiotic use in Zambia | 86 | ****** | yes | yes | yes | yes | yes | not clear | yes |
| Mudenda 2025 | Antimicrobial Stewardship Impact on Antibiotic Use in Three Tertiary Hospitals in Zambia: A Comparative Point Prevalence Survey | 86 | ****** | yes | yes | yes | yes | yes | no | yes |
| | Average | 86 | | | | | | | | |
| | Mode | 86 | | | | | | | | |
| | Max | 86 | | | | | | | | |
| | Min | 86 | | | | | | | | |

**Quality criteria used**

1.1 Are there clear research questions?
1.2 Do the collected data allow to address the research questions?
3.1 Are the participants representative of the target population?
3.2 Are measurements appropriate regarding both the outcome and intervention (or exposure)?
3.3 Are there complete outcome data?
3.4 Are the confounders accounted for in the design and analysis?
3.5 During the study period, is the intervention administered (or exposure occurred) as intended?

Legend: yes (green dot); not clear (yellow dot); no (red dot)

## Quantitative descriptive studies

| Author/Year | Title | MMAT score (%) | Quality | 1.1 | 1.2 | 4.1. | 4.2. | 4.3. | 4.4. | 4.5. |
|---|---|---|---|---|---|---|---|---|---|---|
| Chizimu 2024 | To evaluate the prevalence of antibiotic use and adherence to the World Health Organization (WHO) Access, Watch and Reserve (AWaRe) classification of antibiotics across 16 hospitals in Zambia | 86 | ****** | yes | yes | yes | yes | yes | not clear | yes |
| D'Arcy 2021 | Antibiotic Prescribing Patterns in Ghana, Uganda, Zambia and Tanzania Hospitals: Results from the Global Point Prevalence Survey (G-PPS) on Antimicrobial Use and Stewardship Interventions Implemented | 100 | ******* | yes | yes | yes | yes | yes | yes | yes |
| Kalonga 2020 | Antibiotic Prescribing Patterns in Paediatric Patients at Levy Mwanawasa University Teaching Hospital in Lusaka, Zambia | 100 | ******* | yes | yes | yes | yes | yes | yes | yes |
| Kalungia 2022 | Antibiotic Use and Stewardship Indicators in the First- and Second-Level Hospitals in Zambia: Findings and Implications for the Future | 86 | ******* | yes | yes | yes | yes | yes | not clear | yes |
| Kasanga 2022 | Antibiotic Prescribing Patterns and Prevalence of Surgical Site Infections in Caesarean Section Deliveries at Two Tertiary Hospitals in Lusaka, Zambia | 86 | ****** | yes | yes | yes | yes | yes | not clear | yes |
| Makiko 2024 | Current status and future direction of antimicrobial stewardship programs and antibiotic prescribing in primary care hospitals in Zambia | 100 | ******* | yes | yes | yes | yes | yes | yes | yes |
| Masich 2020 | Antimicrobial usage at a large teaching hospital in Lusaka, Zambia | 71 | ***** | yes | yes | yes | not clear | yes | not clear | yes |
| Miyanda 2022 | Antibiotic prescribing patterns at a level one hospital using national treatment guidelines prescribing indicators in Zambia | 100 | ******* | yes | yes | yes | yes | yes | yes | yes |
| Mudenda 2022 | Antibiotic Prescribing Patterns in Adult Patients According to the WHO AWaRe Classification: A Multi-Facility Cross-Sectional Study in Primary Healthcare Hospitals in Lusaka, Zambia | 100 | ******* | yes | yes | yes | yes | yes | yes | yes |
| Mudenda 2025 | Point Prevalence Survey of Antibiotic Use in Level 1 hospitals in Zambia: Future Prospects for Antimicrobial Stewardship Programs | 86 | ****** | yes | yes | yes | yes | yes | not clear | yes |
| Mudenda 2024 | Hospital prescribing patterns of antibiotics in Zambia using the WHO prescribing indicators post-COVID-19 pandemic: findings and implications | 100 | ******* | yes | yes | yes | yes | yes | yes | yes |
| Mudenda 2023 | Prescribing Patterns of Antibiotics According to the WHO AWaRe Classification during the COVID-19 Pandemic at a Teaching Hospital in Lusaka, Zambia: Implications for Strengthening of Antimicrobial Stewardship Programmes | 100 | ******* | yes | yes | yes | yes | yes | yes | yes |
| Mudenda 2025 | Surveillance of Antibiotic Use and Adherence to the WHO/INRUD Core Prescribing Indicators at a Primary Healthcare Hospital in Southern Zambia: Opportunities for Antimicrobial Stewardship Programs | 100 | ******* | yes | yes | yes | yes | yes | yes | yes |
| Saleem 2025 | Intrapartum and postpartum antibiotic use in seven low- and middle-income countries: Findings from the A-PLUS trial | 86 | ****** | yes | yes | yes | not clear | yes | yes | yes |
| Shawa 2024 | Trends, patterns and relationship of antimicrobial use and resistance in bacterial isolates tested between 2015–2020 in a national referral hospital of Zambia | 86 | ****** | yes | yes | yes | yes | yes | not clear | yes |
| Yamba 2024 | Antibiotic prescribing patterns in primary healthcare facilities and antimicrobial resistance (AMR) profiles of commensal Escherichia coli and enterococci isolated from pregnant women and children under 5 years of age | 86 | ****** | yes | yes | yes | yes | yes | not clear | yes |
| Yasmin 2024 | Antibiotic use in infants in the 6 weeks after delivery in seven low- and middle-income countries: findings from the A-PLUS trial | 86 | ****** | yes | yes | yes | not clear | yes | yes | yes |
| | Average | 92 | | | | | | | | |
| | Mode | 86 | | | | | | | | |
| | Max | 100 | | | | | | | | |
| | Min | 71 | | | | | | | | |

**Quality criteria used**

1.1 Are there clear research questions?
1.2 Do the collected data allow to address the research questions?
4.1. Is the sampling strategy relevant to address the research question?
4.2. Is the sample representative of the target population?
4.3. Are the measurements appropriate?
4.4. Is the risk of nonresponse bias low?
4.5. Is the statistical analysis appropriate to answer the research question?

Legend: yes (green dot); not clear (orange dot); no (red dot)
